# A grid to facilitate physics staffing justification

**DOI:** 10.1120/jacmp.v11i1.2987

**Published:** 2009-12-03

**Authors:** Eric E. Klein

**Affiliations:** ^1^ Department of Radiation Oncology Washington University St. Louis MO USA

**Keywords:** radiotherapy, physics, staffing, levels

## Abstract

Justification of clinical physics staffing levels is difficult due to the lack of direction as how to equate clinical needs with the staffing levels and competency required. When a physicist negotiates staffing requests to administration, she/he often refers to American College of Radiology staffing level suggestions, and resources such as the Abt studies. This approach is often met with questions as to how to fairly derive the time it takes to perform tasks. The result is often insufficient and/or inexperienced staff handling complex and cumbersome tasks. We undertook development of a staffing justification grid to equate the clinical needs to the quantity and quality of staffing required. The first step is using the Abt study, customized to the clinical setting, to derive time per task multiplied by the anticipated number of such tasks. Inclusion of vacation, meeting, and developmental time may be incorporated along with allocated time for education and administration. This is followed by mapping the tasks to the level of competency/experience needed. For example, in an academic setting the faculty appointment levels correlate with experience. Non‐staff personnel, such as IMRT QA technicians or clerical staff, should also be part of the equation. By using the staffing justification grid, we derived strong documentation to justify a substantial budget increase. The grid also proved useful when our clinical demands changed. Justification for physics staffing can be significantly strengthened with a properly developed data‐based time and work analysis. A staffing grid is presented, along with a development methodology that facilitated our justification. Though our grid is for a large academic facility, the methodology can be extended to a non‐academic setting, and to a smaller scale. This grid method not only equates the clinical needs with the quantity of staffing, but can also help generate the personnel budget, based on the type of staff and personnel required. The grid is easily adaptable when changes to the clinical environment change, such as an increase in IMRT or IGRT applications.

PACS number: 87.55.tm, 87.55.Qr

## I. INTRODUCTION

Due to the ever increasing time demands to implement new technologies,^(1)^ justification of clinical physics staffing levels is a daunting and dynamic task. There is also a lack of direction as how to equate clinical needs with the staffing levels and competency required. When a chief physicist negotiates staffing requests to administration, she/he is often directed to the American College of Radiology (ACR) staffing level suggestions as put forth in the Radiation Oncology accreditation program requirements.^(2)^ This ACR publication provides staffing suggestion for all sections within a radiotherapy department. It is a simplistic and often misinterpreted document. Physicists should refer to resources such as the Abt studies.^(3)^ The Abt reports are thorough manpower evaluation documents that detail time (hours) per activity. Neither the ACR document nor Abt reports dissect what level of quality of staff is required. This is unfortunate, especially during time spans when there is a shortage of qualified medical physicists.^(^ ^4^ ^,^ ^5^ ^)^ Instead, the physicist is often met with a simple question: “How much time does it take to perform clinical physics tasks?” The end result is often insufficient and/or inexperienced staff handling complex and cumbersome tasks. Mills et al.^(6)^ published a thorough examination of physics efforts associated with the related billing codes. Purely using cost analysis to justify budgets is risky as billing codes and the reimbursement value may change, often unjustly. An important and thorough method to asses physics staffing levels was conducted by ESTRO (European Society for Therapeutic Radiology and Oncology) and EFOMP (European Federation of Organizations for Medical Physics).^(7)^ This important study led to the development of guidelines by the same organizations for the education and training of radiotherapy physicists.^(8)^ In 2007, we undertook development of a staffing justification grid to equate the clinical needs to the quantity and quality of staffing required.

## II. MATERIALS AND METHODS

### A. Time required for tasks

Our first step was to use the Abt study, customized to our clinical setting, to derive time per task multiplied by the anticipated number of such tasks. We used the “Round II – Final Report” Abt study^(3)^ as guidance for our study. Staffing justification should be inclusive of developmental time. There also must be incorporation of time allocated for education and administration. However, to be conservative, we did not include vacation time, sick time or meeting time for our justification. Non‐faculty personal, such as intensity modulated radiation therapy (IMRT) quality assurance (QA) technicians or clerical staff, if included as part of the physics budget, should also be part of the equation. We detailed all tasks as specific as possible, and subsequently reported the resultant total time for specific tasks over the course of a year. The time per task was extracted from the Abt study values. However, in some cases, we used a slightly lower time/task value, to be conservative. This was partially due to our level of experience, and as a diplomatic gesture to demonstrate to administration that we were being prudent.

### B. Translation of task time to FTE

Upon completing the step of accumulating hours to perform tasks, groupings of particular tasks evolved to consolidate the grid for administrative review. For example, brachytherapy‐related tasks were assessed to collectively be 4160 hours; this would constitute 2.0 FTE based on 2080 hours per FTE.

### C. Experience mapping

The time analysis is followed by mapping the tasks to the level of competency/experience needed. For example, in an academic setting, tasks are mapped to the faculty appointment levels. A higher level of experience and expertise may be necessary for technically challenging tasks related to IMRT and image guided radiation therapy (IGRT). The more routine tasks, such as basic QA, may be carried out by less experienced staff. As previously mentioned, we include non‐faculty personnel as part of the staffing justification. We employ a variety of different personnel to carry out particular tasks. For example, we have a category of staffing called Clinical Physics Assistants (CPA). These are often engineers with Bachelor degrees, or individuals with information technology (IT) expertise. Though they work full time, by virtue of the fact they are funded at approximately half the level of a faculty physicist, we scale their efforts to physics faculty full‐time equivalent (FTE) equivalent by 50%. As our FTE eventually translates to a budget request, it is necessary to scale FTE for non‐Faculty by cost rather than actual hours. In addition, we employ technical staff (often Bachelor degree candidates) to perform IMRT QA. We typically employ three to four individuals who work in the evenings, often two to three simultaneously. Collectively, they work approximately 70 hours per week. These individuals are paid at a rate of approximately 10% of a physics faculty member. Therefore their efforts are scaled accordingly.

### D. Other personnel

Other staff involved peripherally to physics activities including dosimetrists, physics residents, engineers, IT support personnel, and clerical staff, are not supported by our clinical physics contract and therefore are not accounted for in terms of our staff or budget. Depending on the requirements and budget model of a particular facility, staff appropriation and task assignment may need to be included in a staffing assessment. For example, if dosimetrists are incorporated as part of the physics staff and budget, they and their tasks need to be included. The staffing assignment of physics residents will likely vary among institutions. Some residency programs are funded as part of the physics contract and may need to be included. However, training of physics residents does take faculty time, especially in the residents’ first year. This faculty effort needs to be accounted for. Subsequently, if residents are performing billable tasks independently in their second year, this must be accounted for.

**Figure 1:**
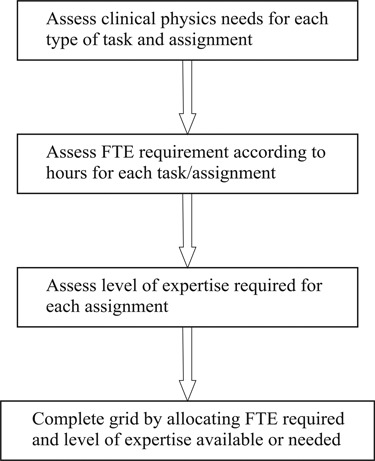
Flowchart of steps involved to generate needed FTE according to tasks and assignments.

The flowchart in Fig. 1 depicts the general sequential steps on how to examine tasks and assignments in order to generate the staffing justification in terms of quantity and expertise.

## III. RESULTS

The assignment of the time values for external beam activities (Section A, below), brachytherapy (B), quality assurance (C), special procedures (D), imaging (E), IMRT (F), education (G), administration (H), computer support (I), developmental time (J), and gamma knife radiosurgery (K), are listed in the section and Tables that follow. The values are based on our activities in 2007. Some of the time values came from the Abt study; others were derived from consensus opinion of our clinical physics staff. Billable activities yielded the number of events for most tasks.

## A. External beam services

### A.1 Routine external beam tasks

Based on 1400 conventional patients per year, the hours are spent to accomplish general routine tasks, as listed in Table 1.

**Table 1:** Routine external beam tasks and time.

*Task*	*Time per Task (hr)*	*No. of Events per Year*	*Total Time (hrs) per Year*
Chart Review	0.20	1400×5(weeks per patient)=7,500	1,400
Basic Calculation Check	0.10	4410	441
Simple Conventional Treatment Plan (TP) (consult and check)	0.4	560	224
Complex Conventional TP (consult and check)	1.0	339	339
In‐vivo Dosimetry	0.1	630	63
Special Medical Physics Consultation (for non‐special procedures)	2.5	140	350
Special Dosimetric Measurements	1.00	50	50
Total Hours			2867

### A.2 Unscheduled consultations

This effort totals 625 hours annually. We summed the special medical physics consultations (that were not part of a special procedure to not double account) and estimated the time expended for each. For example, a pacemaker inquiry involving dosimetric evaluation constitutes five hours per inquiry. Other examples include peripheral dose assessment (~5hours), the rare pregnant patient investigation (25 hours), surface dose assessment (2.5 hours).

### A.3 Construction and/or verification of special bolus/immobilization devices or compensators

This effort totals 80 hours annually.

### A.4 Room remodeling and equipment specifications

This effort totals approximately 200 hours annually.

## B. Brachytherapy

The brachytherapy service is one of the busiest in the world, covering a multitude of procedures which are detailed below (including billing codes when appropriate).

### B.1 LDR brachytherapy

The physics group spends approximately 2.0 hours per implant. This effort totals 200 hours annually to cover 100 implants.

### B.2 HDR brachytherapy (*billing codes 77300, 77336, 77370, 77263, etc*.)

125 GYN, esophageal, sarcoma cases per year (average of 6 procedures/patient), and 50 cases per year for breast HDR. On average, 12 hours per patient is spent, accounting for multiple fractions, for which there are 10 for breast patients. This effort totals 1500 hours annually to cover 1250 HDR procedures. The main tasks include daily QA, review of plans and transferred data, case attendance, and surveys.

### B.3 Radiopharmaceuticals (*77263, 77336, 77300, etc*.)

160 cases per year. This effort totals 320 hours annually to cover 160 procedures.

### B.4 Eye plaque (*77379, 77263, 77328, etc*.)

50 cases per year. This very special procedure takes 5 hours per case. This effort totals 250 hours annually to cover 50 procedures, mainly based on planning and case attendance.

### B.5 Hyperthermia (*77600, 7737, etc*.)

This effort totals 180 hours annually to cover 15 procedures.

### B.6 Prostate seed implants (*77263, 77370, 77371, etc*.)

This effort totals 350 hours annually to cover 50 procedures. Activities associated with this work includes pre‐planning, seed ordering, receipt, assay, preparation, OR attendance, radiation survey, patient instruction, and post‐planning.

### B.7 Intravascular brachytherapy (*77300, 77370, etc*.)

This effort totals 200 hours annually to cover 100 procedures.

### B.8 Brachytherapy inventory maintenance

This effort totals 260 hours annually and is comprised of quarterly inventories and coordination of shipping.

### B.9 Radiation safety

This effort totals 208 hours annually, mainly for annual education of therapy personal and on‐going nursing education.

### B.10 Other brachytherapy hours

Total hours for equipment evaluation/specification, room design/shielding, dosimetry equipment calibration/maintenance (see prior descriptions) is 550 hours annually.

Total brachytherapy service hours=4,018 to cover 1410 procedures. This equates to a coverage need of approximately 2.0 FTE for the 1410 procedures, which yields a ratio of 705 brachytherapy procedures / FTE.

## C. Quality assurance

### C.1 Quality assurance of all treatment machines, simulators, CT‐simulators, treatment planning systems

This effort totals 1265 hours annually. This is based on time estimates for weekly, monthly, and annual reviews as performed on our accelerators and simulator devices, and the maintenance and calibration of all dosimetry measurement equipment.

## D. External beam special procedures (with related billing codes listed)

### D.1 Linac cranial radiosurgery (*77315, 77300, 77370, etc*.)

Estimated for 12 patients per year, ased on 8 hours per case. (The Abt survey estimates 8 hours per patient case.) This effort totals 96 hours annually.

### D.2 Stereotactic body frame (SBF) radiotherapy

Based on 40 patients per year – 400 hours for patient support. This effort totals 400 hours annually based on 10 hours per case to perform plan review, review the treatment execution, and attendance at each fraction.

### D.3 Total body irradiation (TBI) (*77336, 77300, etc*.)

On average, 150 patients per year, with 2.0 hours per case spent. (The Abt study estimated 5.2 hours per patient.) This effort totals 300 hours annually.

The composite total for special procedures is 796 hours.

## E. Imaging

### E.1 Imaging service for treatment planning support

This includes the ultrasound systems, conventional simulator, and CT‐simulators. This effort total 850 hours annually. The majority of this time is attributed to equipment and procedure development and training.

### E.2 Imaging service for treatment localization

This effort totals 850 hours annually and includes devices such as electronic portal imaging devices (EPID), on‐board kV imagers, and video surface imaging devices. The majority of this time is attributed to equipment and procedure development, case attendance, training and also evaluation, and implementation of vendor‐provided equipment and software.

## F. IMRT Patient activities (*77301, 77418*)

Average of 600 patients per year based on trend of 12 new starts per week. On average, 9 hours per patient is spent. (Abt study estimates 12.6 hours per patient). This effort totals 5400 hours annually. These activities include review of plans on‐screen, generation of QA plans, review of the performed QA, and review of parameters to be used for treatment.

## G. Education

Our facility has teaching programs for medical residents, physics residents, therapy and dosimetry students, and summer medical students.

### G.1 Training and continuing education

This effort totals 874 hours annually, which is equivalent to 0.42 FTE. Most of this effort is attributed to in‐services and ongoing education for technical staff members.

### G.2 Classroom teaching

This effort totals 368 hours annually, which is equivalent to 0.18 FTE.

This totals a need for 0.5 FTE for teaching and education.

## H. Administrative support

Our medical physics division has a director, and some administrative duties are performed by chiefs of clinical physics and physics education. This effort totals 1404 hours annually, which is equivalent to 0.70 FTE. The administrative duties include such tasks as faculty development and evaluation, scheduling and organization, and meetings with faculty and other department directors.

## I. Computer support

This effort totals 624 hours annually – 416 hours for external beam, and 208 hours for IMRT. This effort involves management of TP systems and R&V systems that fall outside of the hospitals IT support.

## J. Developmental time

One important negotiating point with the hospital was to appropriate 20% developmental time for each faculty member. This was agreed upon for the purpose of faculty having dedicated time to develop new clinical procedures and work on implementation of new equipment. In addition, the effort of some faculty totals less than 1.0 FTE. This accounts for those who are partially funded by grants.

## K. Gamma knife radiosurgery (GKR)

On average, 240 patients per year, based on 6.25 hours per case. (Abt survey estimates 8 hours per patient case.) This is equivalent to roughly 0.75 FTE (not including administration). (Note: Physicists function as Authorized Medical Physicists and also perform the treatment planning for GKR (77315, 77300, 77370, etc.)).

## L. Comparison with ACR values

Our total external beam service hours (which includes FTE for external beam services, QA, special procedures, IMRT, imaging, IT support, and gamma knife) equals 14,614 hours, the time required to handle 1400 conventional patients, 600 IMRT patients, as well as TBI and radiosurgery patients. This equates to 2030 total external beam patients covered (according to the simplistic ACR approach of patients per FTE) by 7.00 FTE which yields a ratio of: 300 external beam patients (30% IMRT) / Physics Faculty FTE. According to the ACR recommendations^(2)^ of 333 patients per physicist, only 6.0 FTE would be required for our external beam services, with no appropriation for level of expertise. In addition, our 7.0 FTE does not (yet) include time for education, administration, and developmental efforts (and additional 3.4 FTE) which is assumed imbedded within the ACR values. Therefore, using the ACR ratio would have left us with 4.4 FTE less than the model proposed here for all external beam services. (Brachytherapy is not discussed in the ACR FTE ratios.)

Assignment of FTE as calculated by the hours and broken down by tasks is found in Table 2. This lead to the final grid based on 2007 activities as shown in Table 3. As observed, the grid distributes the total FTE according to tasks, and the appropriation of the proper level of expertise for the tasks. As our facility is academic, the expertise level increases from the Instructor level to the Assistant Professor to the Associate Professor. As an example, one particular Associate Professor is assigned 30% time for IMRT and 20% for imaging related to TP. One particular instructor spends 30% time on conventional external beam, and 30% time on brachytherapy.

**Table 2:** Assignment of Tasks to Hours to FTE.

*Section*	*Task*	*Hours*	*FTE*
A	External Beam Services	3750	1.75
B	Brachytherapy	4018	2.0
C	QA	1265	0.6
D	Special Procedures	796	0.25
E‐1	Imaging for TP	850	0.4
E‐2	Imaging for Treatment	1150	0.55
F	IMRT	5400	2.5
G	Education	1312	0.5
H	Administration^a^	1404	0.7
I	IT Support	925	0.5
J	Developmental Efforts		2.2
K	Gamma Knife	1500	0.75
	TOTALS		12.7

^a^ Administrative 0.5 FTE: Physics Director

**Table 3:** Comprehensive grid for staffing levels and expertise appropriation.

*Section*	*Task/Person*	*Director*	*Assoc*	*Assoc*	*Assoc*	*Assoc*	*Assist*	*Assist*	*Instruct*	*Instruct*	*Instruct*	*Instruct*	*Instruct*	*CPAs* ^b^	*Total*
A	External Beam Services		0.1	0.05	0.05	0.05	0.05	0.05	0.2	0.1	0.25	0.05	0.3	0.5	1.75
B	Brachytherapy			0.4	0.1		0.2	0.15		0.3	0.2	0.1	0.3	0.25	2
C	QA		0.05	0.05	0	0.1	0.05	0.05	0.05	0.05	0.05	0.05	0.05	0.05	0.6
D	Special Procedures		0.05			0.1			0.05					0.05	0.25
E‐1	Imaging for TP				0.2			0.15				0.05			0.4
E‐2	Imaging for Treatment		0.05				0.1	0.15	0.1			0.15			0.55
F	IMRT	0.05	0.2	0.25	0.3	0.1	0.25	0.2	0.15	0.1	0.1	0.35	0.15	0.35	2.5
G	Education		0.05	0.05	0.05	0.05	0.05	0.05	0.05	0.05	0.05	0.05			0.5
H	Administration^a^	0.5^a^	0.2												0.7
I	IT Support													0.5	0.5
J	Developmental Efforts		0.2	0.2	0.2	0.2	0.2	0.2	0.2	0.2	0.2	0.2	0.2		2.2
K	Gamma Knife		0.1		0.1	0.25	0.1			0.2					0.75
	TOTALS	0.55	1	1	1	0.85	1	1	0.8	1	0.85	1	1	1.7	12.7

^a^ Administrative 0.5 FTE: Physics Director

^b^ Current CPA (Clinical Physics Assistants) Utility: IT Support ‐ IT Specialist (scaled to 50% salary of instructor); 170 hours/week for various engineering support, mainly imaging related (scaled to 50% of Instructor salary); 70 hours/week for IMRT QA (scaled to 10% of Instructor salary)

1FTE=2080hours.

External Beam Services includes conventional external beam services & tasks, QA, consultations, IT support, equipment evaluation, room design, equipment maintenance/calibration)).

Imaging for TP: CT Simulation, PET, MR, Image Fusion.

Imaging for Treatment: Daily Localization Devices.

Special Procedures include TBI, ESRT, Linac Radiosurgery (includes SRS, ESRT, TBI).

Assoc: Associate Professor Level.

Assist: Assistant Professor.

Instruct: Instructor.

As an example of the breakdown of tasks for a given physicist for a particular assignment, we use IMRT. The IMRT service is covered in total of 2.5 FTE by faculty members and CPAs. We focus on the three physicists who have the greatest amount of their time assigned to IMRT. The IMRT tasks for these particular physicists are detailed as follows:

- Associate Professor (0.3 FTE): Primary responsibility is for commissioning and overall supervision of one particular IMRT planning and delivery system. In addition, provides coverage one day/week for the IMRT planning service by advising dosimetrists with final review and approval of completed plans.
- Assistant Professor (0.25 FTE): Provides coverage one day/week for the IMRT planning service by advising dosimetrists with final review and approval of completed plans. In addition, is primarily responsible for management of IMRT patient QA, ensuring which IMRT measurements are performed and when.
- Instructor (0.35 FTE): Provides coverage one day/week for the IMRT planning service by advising dosimetrists with final review and approval of completed plans. In addition, performs the majority of QA plans for later comparison with measurements provided by the CPAs.

Other physicists provide IMRT coverage of plan review, QA, and commissioning.

A summary of our mapping assigning particular FTE to tasks in found in Table 3.

## IV. DISCUSSION

By using the staffing justification grid, we derived strong documentation to justify a substantial budget increase. Prior to this successful request using the grid, we were performing approximately the same procedures with only 9 FTE. Since the time this grid was completed, we have had two changes to our clinical operation. The percentage of patients receiving IMRT patient has doubled, subsequently reducing our conventional external beam efforts proportionately. As the FTE required for IMRT is substantially higher than for conventional treatment, we require a further increase in staffing. Essentially, the IMRT staffing requirement has doubled from 2.5 FTE to 5.0 FTE (an increase of 2.5 FTE), while the conventional FTE requirement has decreased by 0.75 FTE. In addition, the daily localization procedures have increased dramatically with the introduction of new systems (AlignRT, Calypso, on‐board imaging, cone‐beam CT), increasing this FTE requirement by another 0.25 FTE. Therefore, a total increase of 2.0 FTE is needed. Because IMRT procedures have become more routine, the time required could be equally distributed according to expertise, but the increase in imaging for treatment would be weighted for physicists with more experience. In Table 4 we demonstrate how the FTE will be increased and redistributed based on these changes. As the actual individuals would have to be hired following administrative approval, we denote proposed faculty as placeholders in terms of assignment.

**Table 4:** Comprehensive grid for staffing levels and expertise appropriation for proposed increase.

*Sect*.	*1*	*2*	*3*	*4*	*5*	*6*	*7*	*8*	*9*	*10*	*11*	*12*	*13*	*14*	*15*	*Total*
A							0.05	0.05	0.1		0.1	0.05	0.05	0.1	0.5	1.00
B			0.4	0.1			0.2	0.15			0.3	0.2	0.1	0.3	0.25	2.00
C		0.05	0.05			0.1	0.05	0.05	0.05		0.05	0.05	0.05	0.05	0.05	0.6
D		0.05				0.1			0.05						0.05	0.25
E‐1				0.2				0.15					0.05			0.4
E‐2		0.05			0.25^a^		0.1	0.15	0.1				0.15			0.8
F	0.05	0.2	0.30	0.35	0.75^a^	0.1	0.25	0.2	0.25	1.0^a^	0.1	0.1	0.35	0.15	0.35	5.0
G		0.05	0.05	0.05		0.05	0.05	0.05	0.05		0.05	0.05	0.05			0.5
H	0.5	0.2														0.7
I															0.5	0.5
J		0.2	0.2	0.2		0.2	0.2	0.2	0.2		0.2	0.2	0.2	0.2		2.2
K		0.1		0.1		0.25	0.1				0.2					0.75
Total	0.55	0.9	1	1	1	0.8	1	1	0.8	1	1	0.75	1	0.8	1.7	14.5

^a^ Represents the increased FTE and desired qualifications for the added FTE (2 in total).

Column Legend:

1‐ Director; 2‐ Assoc,; 3‐ Assoc.; 4‐Assoc.; 5‐Assoc. (proposed); 6‐Assoc.; 7‐Assist.; 8‐Assist.; 9‐Instructor; 10‐Instruct. (proposed); 11‐Instruct.; 12‐Instruct.; 13‐Instruct.; 14‐Instruct.; 15‐CP Assist.

The above analysis applies most strictly to an academic practice. An example of the grid for a small, non‐academic clinic is presented in Table 5. This particular clinic possesses three linear accelerators (two providing IMRT), and brachytherapy limited to 125 HDR procedures annually. Approximately 700 conventional and 300 IMRT patients are treated annually. A CT‐simulator is present in the department, and imaging for treatment is limited to one online portal imager. No special procedures or teaching is performed at this particular facility. The resultant FTE of 3.25 is best appropriated by two experienced physicists – one newly certified physicist, plus assistance (the 0.25 FTE) by clinical physics assistant(s) primarily responsible for IMRT QA plans and measurements.

**Table 5:** Grid for staffing level and expertise for small non‐academic facility.

*Task/Person*	*Physicist 1 (Director)*	*Physicist 1 (Very Experienced)*	*Physicist 1 (Newly Certified)*	*CPA (QA Tech) scaled*	*Total*
XRT		0.3	0.5		0.80
Brachy		0.2	0.05		0.25
QA		0.1	0.2		0.30
Imaging TP	0.2				0.20
Imaging Rx	0.1				0.10
IMRT	0.5	0.3	0.25	0.25	1.30
Administrative	0.2				0.20
IT Support		0.1			0.10
Totals	1.00	1.00	1.00	0.25	**3.25**

Note: CPA performs QA plans and QA measurements for IMRT patients.

As our staffing justification heavily emphasizes that a high level of expertise is necessary for particular tasks, it is imperative that we ensure the physicists assigned to those tasks are proficient. We therefore utilize a robust cross‐training program. For example, in order to provide coverage for gamma knife stereotactic radiosurgery, a physicist being trained is shadowed for a minimum of five cases, and must demonstrate proficiency with emergency procedures and the ability to independently perform quality assurance. Similar mentoring takes place for HDR and IMRT coverage. In addition, if a gap of clinical activity occurs (for example, if a physicist covers a minimal number of gamma knife cases over the course of a year), retraining would take place. And finally, if new procedures or equipment is introduced, formal in‐services with mandatory attendance is provided.

## V. CONCLUSIONS

We describe a workload‐driven methodology for justifying physics staffing. Though our detailed grid example is for a large academic facility, the methodology can be extended to a non‐academic setting and to a smaller scale. This grid method not only equates the clinical needs with the quantity of staffing, but it also generates the personnel budget, based on the type of staff and personnel required. The grid is easily adaptable when changes to the clinical environment change, such as an increase in IMRT or IGRT applications.
